# The Frontier Doctor

**Published:** 1875-12

**Authors:** 


The Frontier Doctor.—Few physicians appreciate the toils of professional life on the frontier. A correspondent of an exchange, traveling through the Indian Territory, thus describes one he had met on his journey: A day's ride beyond the settlements, half way between Brownwood and the Colorado, we met a frontier M.D. We knew his profession from his Æsculapian air; he rode a horse of speed and strength, that shied around us with watchful eyes, distended nostrils, and ears apitch. A rifle hung at the saddle, and the doctor merely glanced at us. So far from home, any sort of recognition would have given comfort. We would have bowed profoundly to a pharmaceutical nod of the head, but he rode on, solitary and unsocial, with sealed lips, and as erect as a Cossack. We had the curiosity, subsequently, to ask, and learned that the call was fifty miles distant, through an Indian country, involving a hundred mile ride, not an unfrequent occurrence. The M.D.'s bill, our informant told us, would not exceed fifty dollars. If true, we know not which most to admire, the moderation of the charge, or the courage of the rider.—*The Medical and Surgical Reporter.*

